# Supplementary material for: High-Throughput and Cost-Effective Characterization of Induced Pluripotent Stem Cells
Source: Stem Cell Reports. 2017 Apr 6;8(4):1101–11. doi: 10.1016/j.stemcr.2017.03.011 (PMC5390243; doi:10.1016/j.stemcr.2017.03.011)
Supplement: Document S1. Supplemental Experimental Procedures and Figure S1 [file mmc1.pdf]

Stem Cell Reports, Volume *8*

Supplemental Information

High-Throughput and Cost-Effective Characterization of Induced Pluripotent Stem Cells

Matteo D'Antonio, Grace Woodruff, Jason L. Nathanson, Agnieszka D'Antonio-Chronowska, Angelo Arias, Hiroko Matsui, Roy Williams, Cheryl Herrera, Sol M. Reyna, Gene W. Yeo, Lawrence S.B. Goldstein, Athanasia D. Panopoulos, and Kelly A. Frazer

Supplemental information includes Figure S1, Tables S1-S6 and Data S1.

Supplemental Figure

Figure S1. Differences between low- and high-quality barcoded iPSCs, related to Figure 3.

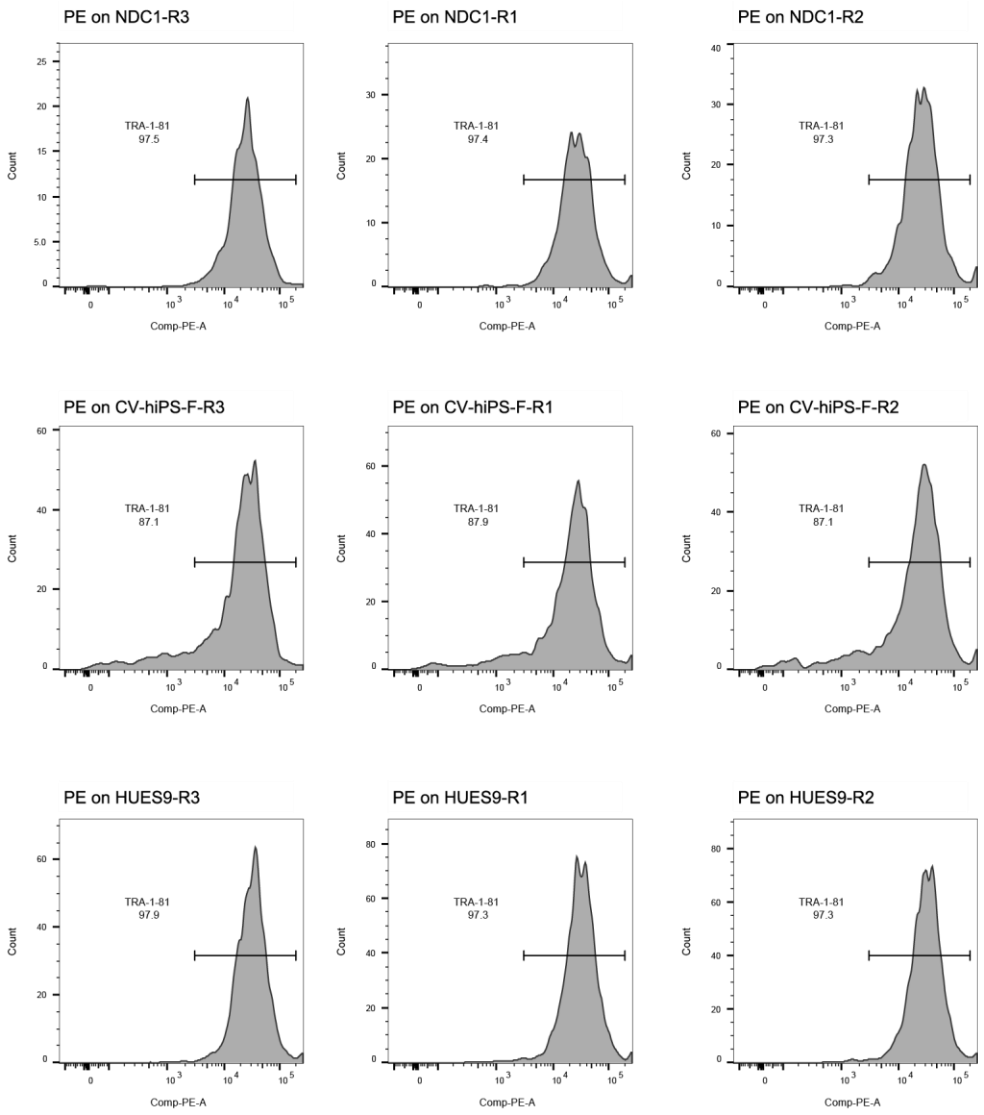

Shown are the histograms of TRA-1-81$^+$ cells for the three replicates of the three lines analyzed in Figure 3. A lower percentage of cells staining positive for TRA-1-81$^+$ in the CV-hiPS-F line (middle row) is in agreement with previous characterization of its quality (Gore et al., 2011), and confirms that FCB allows the distinction between low- and high-quality iPSCs.

Supplemental Tables

Table S1. Description of all lines used for qPCR, related to Figure 4.

The table shows the list of 122 lines used for qPCR. Column C shows the 12 samples that did not pass QC (five iPSCs and one EB, as well as their associated five EBs and one iPSC line) and were not included in PCA (Figure 4).

Table S2. List of primers used for qPCR, related to Figure 4.

Shown is the list of all 68 primer pairs (4 housekeeping genes and 64 pluripotency or germ layer markers) used for qPCR. For each primer pair, the lineage where its associated gene is expressed is displayed (column B). Column C shows the 12 primer pairs that were selected for 12-gene qPCR to calculate each germ layer scores (Figure 4).

Table S3. Ct values detected by qPCR, related to Figure 4.

Shown are the raw Ct values of all 68 primer pairs in the 110 samples that underwent qPCR and passed all quality filters and were used for PCA.

Table S4. Genomic position of all variants in trisomic chromosomes, related to Figure 5.

Shown is the probe ID assigned by Illumina and the genomic position for all 50,076 probes in the trisomic chromosomes (12, 13, 14, 17, 20).

Table S5. Log r ratio for all variants in trisomic chromosomes, related to Figure 5.

Shown is the log r ratio associated with the probes described in Table S4 for CV-hiPS-B, CV-hiPS-F and the serial dilutions.

Table S6. B allele frequency for all variants in trisomic chromosomes, related to Figure 5.

Shown is the B allele frequency associated with the probes described in Table S4 for CV-hiPS-B, CV-hiPS-F and the serial dilutions.

Supplemental Data

Data S1. Calculation of pluripotency and germ layer scores, related to Figure 4.

This Excel sheet allows the user to calculate pluripotency and germ layer scores by providing raw Ct values of 17 genes (four pluripotency markers, four markers for each germ layer and one housekeeping gene) as input. The 12 genes for the three germ layer scores were chosen based on analyses in this manuscript (Figure 4). The four pluripotency markers are from our previous publication (Gore et al., 2011; Liu et al., 2011).

To run the analysis, users will need to: 1) enter sample names in cells A4-A99 (highlighted in blue); 2) enter gene names in cells B3-R3 (highlighted in blue); 3) enter the raw Ct values for the housekeeping gene to be used for normalization in cells B4-B99 (highlighted in green); and 4) enter raw Ct values for the marker genes in cells C4-R99 (highlighted in yellow).

Columns T-AJ show ΔCt values, calculated as $\Delta Ct_{gene} = 2^{(Ct_{housekeeping} - Ct_{gene})}$. Columns AL-BB show normalized expression levels (to values included between zero and one), calculated as $e_{gene} = \frac{(\Delta Ct_{gene} - \min(\Delta Ct_{gene}))}{(\max(\Delta Ct_{gene}) - \min(\Delta Ct_{gene}))}$. The output scores are shown in cells BD2-BH99, while the corresponding heatmap is shown in cells BJ2-BN99.

For demonstrative purposes, two example datasets are provided for the user to test the Data S1 Excel sheet. Dataset 1 is provided to test calculating a pluripotency score, the Excel sheet is populated with raw Ct values from five samples (including two ESCs, two iPSCs and one fibroblast) that have been previously characterized (Gore et al., 2011; Liu et al., 2011). Dataset 2 is provided to test germ-layer scores, the Excel sheet is populated with raw Ct values from nine samples (three ESCs, three iPSCs and their associated EBs) generated in this manuscript.

Below are the two example datasets provided for the user to test the Data S1 Excel sheet:

Dataset 1: Five samples assessed for four pluripotency markers (Gore et al., 2011; Liu et al., 2011). The user pastes these sample names, gene names and raw Ct values of each gene into Data S1 Excel sheet.

Sample name	Housekeeping gene	Pluripotent			
	GAPDH	End-OCT4	End-SOX2	NANOG	CRIPTO
H1 (ES Line)	14.500	18.820	18.000	19.000	17.000
H9 (ES line)	14.670	17.000	18.000	19.000	17.000
FiPS4F2 (iPS line)	13.950	18.000	18.000	20.000	17.000
FiPS4F5 (iPS line)	15.860	19.000	20.000	20.000	18.000
IMR90 (Fibroblasts)	14.500	23.000	31.000	27.000	29.000

Dataset 2: Nine samples with 12 germ layer markers (four for each germ layer). The user pastes these sample names, gene names and raw Ct values of each gene into Data S1 Excel sheet.

Sample name	Housekeeping gene	Ectoderm			
	RPS29	ALDH1A1	COL1A1	PAX6	DCX
ES.HUES16 p50	2.500	13.310	8.925	13.901	14.302
ES.HUES9 p39	3.364	15.790	10.500	20.077	14.460
ES.H9 p54	2.500	14.285	11.136	16.280	13.530
IPS.F2.11	3.146	14.796	9.321	13.168	15.250
IPS.F2.24	3.811	16.486	8.934	14.849	15.706
IPS.F1.21	3.728	15.662	8.790	14.514	15.975
EB.F1.21	4.771	13.870	8.422	15.564	15.442
EB.F2.11	4.537	11.970	8.011	14.046	15.820
EB.F2.24	5.323	14.977	9.229	15.074	17.497

Sample name	Housekeeping gene	Endoderm			
	RPS29	AFP	GATA1	SOX7	CDH1
ES.HUES16 p50	2.500	13.320	20.451	16.000	12.768
ES.HUES9 p39	3.364	25.800	20.192	16.588	13.490
ES.H9 p54	2.500	999.000	23.679	14.778	12.333
IPS.F2.11	3.146	16.445	999.000	16.368	12.778
IPS.F2.24	3.811	18.758	21.379	17.129	12.580
IPS.F1.21	3.728	13.694	21.679	17.002	12.999
EB.F1.21	4.771	5.948	20.712	14.518	12.844
EB.F2.11	4.537	3.869	16.835	13.737	11.062
EB.F2.24	5.323	6.852	999.000	14.907	11.805

Sample name	Housekeeping gene	Mesoderm			
	RPS29	DCN	GATA2	BMP4	IGF2
ES.HUES16 p50	2.500	14.114	15.979	11.766	15.107
ES.HUES9 p39	3.364	21.549	17.743	13.597	17.962
ES.H9 p54	2.500	20.024	17.257	13.560	19.049
IPS.F2.11	3.146	13.128	16.635	12.154	13.227
IPS.F2.24	3.811	23.982	17.141	13.781	16.442
IPS.F1.21	3.728	19.900	14.740	11.410	14.250
EB.F1.21	4.771	9.046	13.037	12.312	8.908
EB.F2.11	4.537	9.188	12.863	12.255	9.764
EB.F2.24	5.323	10.275	12.584	13.954	10.119

References

Gore, A., Li, Z., Fung, H.L., Young, J.E., Agarwal, S., Antosiewicz-Bourget, J., Canto, I., Giorgetti, A., Israel, M.A., Kiskinis, E., *et al.* (2011). Somatic coding mutations in human induced pluripotent stem cells. Nature *471*, 63-67.

Liu, G.H., Barkho, B.Z., Ruiz, S., Diep, D., Qu, J., Yang, S.L., Panopoulos, A.D., Suzuki, K., Kurian, L., Walsh, C., *et al.* (2011). Recapitulation of premature ageing with iPSCs from Hutchinson-Gilford progeria syndrome. Nature *472*, 221-225.
