# Supplementary material for: High-Throughput and Cost-Effective Characterization of Induced Pluripotent Stem Cells
Source: Stem Cell Reports. 2017 Apr 6;8(4):1101–11. doi: 10.1016/j.stemcr.2017.03.011 (PMC5390243; doi:10.1016/j.stemcr.2017.03.011)
Supplement: Document S2. Article plus Supplemental Information [file mmc9.pdf]

Stem Cell Reports
Resource
OPEN ACCESS

High-Throughput and Cost-Effective Characterization of Induced Pluripotent Stem Cells

Matteo D'Antonio,[1] Grace Woodruff,[2] Jason L. Nathanson,[2] Agnieszka D'Antonio-Chronowska,[3] Angelo Arias,[1] Hiroko Matsui,[3] Roy Williams,[3] Cheryl Herrera,[2] Sol M. Reyna,[2] Gene W. Yeo,[2,3] Lawrence S.B. Goldstein,[2,4,*] Athanasia D. Panopoulos,[5,**] and Kelly A. Frazer[1,3,***]

[1]Department of Pediatrics
[2]Department of Cellular and Molecular Medicine
[3]Institute for Genomic Medicine
[4]Department of Neurosciences
University of California, San Diego, La Jolla, CA 92093, USA
[5]Department of Biological Sciences, University of Notre Dame, Notre Dame, IN 46556, USA
*Correspondence: lgoldstein@ucsd.edu (L.S.B.G.), apanopou@nd.edu (A.D.P.), kafrazer@ucsd.edu (K.A.F.)
http://dx.doi.org/10.1016/j.stemcr.2017.03.011

SUMMARY

Reprogramming somatic cells to induced pluripotent stem cells (iPSCs) offers the possibility of studying the molecular mechanisms underlying human diseases in cell types difficult to extract from living patients, such as neurons and cardiomyocytes. To date, studies have been published that use small panels of iPSC-derived cell lines to study monogenic diseases. However, to study complex diseases, where the genetic variation underlying the disorder is unknown, a sizable number of patient-specific iPSC lines and controls need to be generated. Currently the methods for deriving and characterizing iPSCs are time consuming, expensive, and, in some cases, descriptive but not quantitative. Here we set out to develop a set of simple methods that reduce cost and increase throughput in the characterization of iPSC lines. Specifically, we outline methods for high-throughput quantification of surface markers, gene expression analysis of in vitro differentiation potential, and evaluation of karyotype with markedly reduced cost.

INTRODUCTION

A crucial problem in both the analysis of many human diseases and the development of effective therapies to treat disease is the incomplete understanding of the role played by human genetic variation in their development. An important translational tool needed to solve this problem is an in vitro cellular model derived from large numbers of individuals who display both sporadic and inherited disease as well as healthy controls. Pluripotent stem cells can provide disease-relevant cell types to model human diseases. To date, many cell types have been derived from pluripotent cell lines, and exciting advances in disease modeling and drug screening have been published (Avior et al., 2016; Brennand et al., 2011; Israel et al., 2012; Itzhaki et al., 2011; Mertens et al., 2015). However, a current limitation to using induced pluripotent stem cells (iPSCs) to model human disease is the time-inefficiency and cost of standard characterization methods required after reprogramming. Furthermore, to model certain diseases, hundreds of patient-specific pluripotent lines are necessary to be adequately powered to test the relationship of genetic variants with cellular phenotypes and disease development.

Current methods for assessing pluripotency are low-throughput and expensive. With the development of several large biobanks of iPSCs to serve as resources for studying human genetic variation and disease (Kilpinen et al., 2016; McKernan and Watt, 2013; Panopoulos et al., 2017 [this issue of *Stem Cell Reports*]; Salomonis et al., 2016), the need to find low-cost, high-throughput solutions to characterize iPSC pluripotency and genomic integrity has become a high priority. The teratoma assay, which measures human iPSC pluripotency in vivo, requires the injection of iPSCs into immunodeficient mice. This assay is expensive, technically challenging, time consuming, and can be inconsistent in results (Andrews et al., 2015). Embryoid body (EB) formation assays (Kurosawa, 2007) provide a cheaper and less labor-intensive alternative by testing the ability of iPSC lines to differentiate into the three germ layers (mesoderm, endoderm, and ectoderm) in vitro. This method is easily scalable because it does not require addition of growth factors or plating of cells on matrices to induce lineage differentiation, and can be readily performed in a multiwell format. However, neither the teratoma nor EB assays enable one to distinguish between high-quality iPSC lines composed of a high percentage of pluripotent stem cells from those that may be more heterogeneous in nature but that contain a subpopulation of cells that are pluripotent. Therefore, to maximize the ability to utilize hundreds of iPSC lines for genetic studies, researchers need methods to assess both pluripotency and heterogeneity in an efficient manner.

Flow cytometry can assess cell-surface expression of pluripotent markers (e.g., TRA-1-60, TRA-1-81) at the single-cell level and is easily scalable using fluorescent cell barcoding (FCB) (Krutzik and Nolan, 2006). In FCB, each

CrossMark
This is an open access article under the CC BY license (http://creativecommons.org/licenses/by/4.0/).

sample in each well is labeled with a different signature, or barcode, of fluorescent dyes with variable intensities and emission wavelengths. Samples from multiple wells are pooled together prior to staining with antibodies specific for the markers of interest and then analyzed by flow cytometry. This method reduces antibody consumption by 100-fold, eliminates staining variability between samples, and decreases acquisition time per plate to 5–15 min (Krutzik and Nolan, 2006). Importantly, FCB enables one to distinguish between high-quality iPSC lines composed of predominantly pluripotent stem cells from those that are heterogeneous. Conversely, while existing gene expression-based assays, such as PluriTest and TaqMan hPSC Scorecard Assay (Muller et al., 2011; Tsankov et al., 2015), cannot account for heterogeneous cell populations, they can provide an accurate view of pluripotency and the differentiation potential of iPSCs, respectively, based on the expression of a larger number of genes. Therefore, the optimal solution to determine heterogeneity and pluripotency of iPSCs may be a combination of flow cytometry and gene expression assays.

Genomic integrity is also essential to characterize, as somatic copy-number variants (CNVs) could potentially affect cellular function or, in the case of genetic studies, the interpretation of inherited variants. At present, it is standard practice to monitor genomic stability of iPSCs by G-band karyotype analysis, which has allowed the detection of large duplications involving genes that could potentially affect pluripotency and differentiation potential (Maitra et al., 2005; Spits et al., 2008; Wu et al., 2008). This technique is generally performed by trained cytogeneticists in commercial laboratories, is costly, and requires the preparation and shipment of live cells. The resolution of this technique is, at best, limited to a chromosomal rearrangement of 5 Mb or larger (Elliott et al., 2010), and is impractical for high-throughput analysis of iPSCs. We and others initiated the use of SNP microarray technology for the routine karyotyping of iPSCs using arrays such as the Illumina HumanCoreExome BeadChip (International Stem Cell Initiative et al., 2011; Laurent et al., 2011; Mayshar et al., 2010; Panopoulos et al., 2017; Taapken et al., 2011). This method is relatively inexpensive (up to 6-fold cheaper than G-band karyotype analysis), has high sensitivity, and has up to 50-fold better resolution (100 kb) than karyotyping (Hulten et al., 2003; Wapner et al., 2012). Previous studies using SNP arrays for examining genomic integrity, however, have not fully investigated their sensitivity for detecting somatic CNVs in subpopulations of cells in an iPSC line. Therefore, it is still unknown to what extent arrays can detect subclonal chromosomal rearrangements in an iPSC population.

The analysis of available methods suggests that a combination of several assays, including flow cytometry to investigate heterogeneity, gene expression analysis to examine in vitro differentiation potential, and high-resolution karyotyping to detect chromosomal aberrations, is necessary for a complete characterization of iPSC lines. Here, we describe a cost-effective, high-throughput suite of these methods including flow cytometry using FCB, qPCR (based on 12 primer pairs) for expression analysis, and SNP arrays for digital karyotyping (Figure 1), which will facilitate the characterization of the large numbers of iPSC lines currently being generated in individual laboratories as well as in biobanks to examine human diseases.

RESULTS

Reprogramming and Barcoding for Surface Marker Expression

To develop a simple method for initial characterization of reprogrammed cells by flow cytometry, we reprogrammed fibroblasts from eight individuals using retroviruses with a standard *OCT4*, *KLF4*, *SOX2*, and *c-MYC* cocktail (OKSM). Additionally each factor had a GFP tag so that silencing of retroviral factors could be monitored (Chan et al., 2009). We included fibroblasts from two individuals with a familial Alzheimer's disease (FAD) mutation in the amyloid β precursor protein (APP), two non-demented control (NDC) individuals, three individuals with sporadic Alzheimer's disease (SAD), and one individual with hippocampal sclerosis (Table 1) to ensure that our methods would be applicable for analysis of cell lines regardless of disease status. All eight fibroblast lines generated colonies, and a total of 294 individual colonies (range 24–50 colonies per fibroblast line) were manually picked based on morphology (compact, circular) and absence of GFP, indicating that retroviral factors had been silenced (Table 2). Individual colonies were subsequently passaged and expanded for additional characterization.

With traditional characterization methods, each iPSC line was tested individually for expression of pluripotency markers by immunofluorescence or flow cytometry. To accelerate this process, reduce antibody consumption, eliminate staining variability between samples, and decrease the cost of measuring expression of pluripotent markers, we adapted FCB (Krutzik and Nolan, 2006) for use with iPSCs. As depicted in Figure 2, we optimized FCB using three dyes (three concentrations of Alexa 750, four concentrations of Alexa 647, and five concentrations of Pacific Blue) to allow analysis of TRA-1-60 or TRA-1-81 in 60 different iPSC lines simultaneously.

To test whether the FCB technique would be able to distinguish between high- and low-quality iPSCs, we performed a pilot experiment with a human embryonic stem cell (hESC) line (HUES9), a high-quality iPSC line

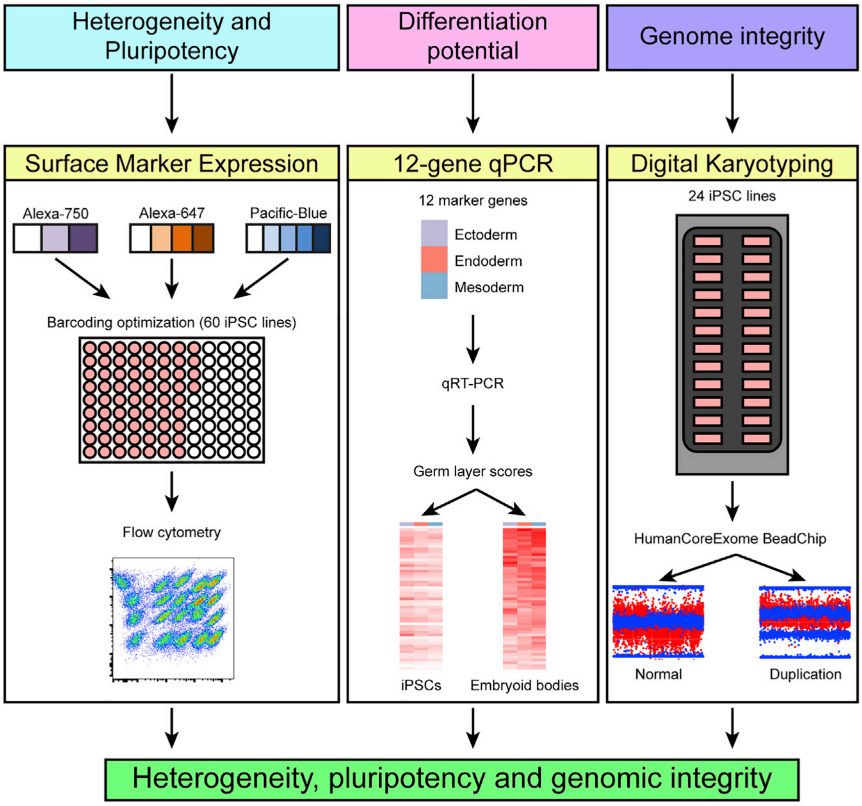

Figure 1. Workflow to Characterize iPSC Lines

Simple and cost-effective methods for determining heterogeneity, differentiation potential, and genome integrity of iPSC lines. Heterogeneity is assessed by flow cytometry on up to 60 iPSC lines simultaneously using barcoding optimization. In vitro differentiation potential is examined by qPCR using 12 marker genes on up to 96 samples. Digital karyotype is determined using Illumina genotyping BeadChips.

(NDC1), and a low-quality iPSC line (CV-hiPS-F). The high-quality iPSC line was previously generated in our laboratory (Israel et al., 2012) and displays high expression of pluripotent markers and the ability to differentiate into the three germ layers. The low-quality iPSC line was also generated in our laboratory (Gore et al., 2011) and was characterized as such due to the presence of GFP-positive (GFP^+) cells (indicating retroviral reactivation), low expression of pluripotent markers, irregular colony morphology, and an abnormal karyotype. Three biological replicates of each cell line were barcoded (Figure 3A), divided into two tubes (with one tube stained with TRA-1-60 and the other stained with TRA-1-81), and analyzed for the presence of GFP^+ cells (Figures 3 and S1). The hESC line and the high-quality iPSC line exhibited no GFP^+ cells (Figures 3B and 3D), while the low-quality iPSC line displayed GFP^+ cells (Figure 3F). In addition, the hESC line and high-quality iPSC line exhibited a higher number of cells positive for TRA-1-81 (>97%) than the low-quality iPSC line (87.4%, $p = 0.025$, Wilcoxon rank-sum test) (Figures 3C, 3E, 3G, and S1). Based on the results from our pilot experiment, it is clear that using FCB to examine the percentage of cells expressing pluripotent markers can distinguish between high- and low-quality iPSCs.

To test whether FCB could support analysis of large numbers of lines, we analyzed iPSC lines from our reprogramming collection by FCB for expression of TRA-1-60 and TRA-1-81, and absence of GFP at passage 3 (P3). From the 294 colonies that were manually picked (Table 2), 162 (~55%) maintained good colony morphology (compact, circular) during expansion and were analyzed by FCB. Of these 162 iPSC lines, 149 (~92%) lines were high-quality lines (i.e., had high expression levels of TRA-1-60 and TRA-1-81 and no GFP^+ cells, as depicted in Figure 3) (Table 2), and were subsequently frozen down for further analysis. Thus, FCB is a highly scalable assay that can efficiently characterize hundreds of iPSC lines.

Twelve-Gene qPCR to Assess In Vitro Differentiation Potential

We sought to establish a quantitative method that was both easy to implement and had a straightforward analysis approach to test whether reprogrammed cells had pluripotent gene expression and an ability to differentiate in vitro. Of the 149 iPSC lines expressing cell-surface pluripotency markers, we chose 58 to test by qPCR for gene expression signatures of pluripotency and the ability to differentiate into the three germ layers (mesoderm, endoderm, and ectoderm). We conducted undirected EB differentiation of these 58 iPSC lines (Table S1) and interrogated 30 markers of pluripotency and markers for each of the three germ layers (9 ectoderm, 8 endoderm, and 17 mesoderm) by

Table 1. Summary of Fibroblast and Subject Information

Fibroblast Name	Family History	Diagnosis	Age at Biopsy	Age of Onset	MMSE[a]	APOE[b]	Sex
APP[c] V717F-1	yes	FAD[d]	unknown	unknown	unknown	3/3	M
APP[c] V717F-2	yes	FAD	unknown	unknown	unknown	3/3	F
NDC4	no	NDC[e]	84	NA	30	3/3	M
NDC5	no	NDC	79	NA	30	3/3	M
SAD3[f,g]	yes	PAD[h]	89	82	23	3/3	M
SAD4	yes	PAD	79	74	24	3/3	F
SAD5	yes	PAD	84	80	26	3/3	F
SAD6	yes	PAD	81	78	24	3/3	M

[a]MMSE, Mini Mental State Examination (Folstein et al., 1975).
[b]Apolipoprotein E (APOE) genotype indicates carriers are homozygous for the ε3 allele.
[c]APP, amyloid precursor protein.
[d]FAD, familial Alzheimer's disease.
[e]NDC, non-demented control.
[f]SAD, sporadic Alzheimer's disease.
[g]Upon autopsy, individual SAD3 was reported to have hippocampal sclerosis, not SAD.
[h]PAD, probably Alzheimer's disease.

qPCR, normalizing the expression data using the housekeeping gene *RPS29* (see Experimental Procedures) (Tables S2 and S3). While conducting a quality check of the qPCR results (measured as "Ct"), six iPSC-EB pairs were removed because one or both samples were found to be of insufficient quality based on the following criteria: (1) *RPS29*, the housekeeping gene used for normalization, was not expressed; (2) less than 50% of the tested genes were expressed; or (3) more than 50% of the tested genes were aberrantly expressed (defined as an expression level greater than two SDs from its mean expression value across all samples after normalization to *RPS29*). Using a principal component analysis (PCA) on the expression profiles of these 64 marker genes, we analyzed 110 samples comprising 52 iPSCs, 52 associated EBs, and six ESCs included as controls (Figures 4A–4E), and confirmed that the expression levels of these genes were able to distinguish between pluripotent and differentiated cell lines.

To make the qPCR method as cost-effective and streamlined as possible, we next analyzed the 64 marker genes to choose an optimal set of 12 markers that would be sufficient to establish the differentiation potential of iPSCs. To achieve this, we took advantage of the fact that iPSCs were found to be associated with low values of principal component 2 (PC2) and PC3, whereas EBs had low values of PC2 and PC3 (Figures 4A–4C). We chose four genes per germ layer with the most negative weights on PC2 (Figure 4D). We then determined how well these 12 markers were able to detect the presence of the three germ layers in the derived EBs. Expression levels for the 12 genes were normalized across all 110 samples (minimum = 0 and maximum = 1). For each germ layer, we defined a "germ layer score" as the mean normalized expression values of the four genes most associated with each germ layer. We found that the ESCs and iPSCs have very low germ layer scores, while the majority of the EBs have high scores for all three germ layers (Figure 4E). As expected (Tsankov et al., 2015), the relative strength of the three germ layer scores varied across the EBs, suggesting that they had different proportions of ectoderm, endoderm, and mesoderm cells. These data suggest that the in vitro differentiation potential of iPSC can be efficiently examined by assaying derived EBs for expression of these 12 genes. To make pluripotency and multilineage differentiation analysis accessible, we include a supplementary file, Data S1, a Microsoft Excel table that allows users to generate pluripotency and/or germ layer scores and corresponding heatmaps using Ct values of a housekeeping gene and marker genes of interest.

Digital Karyotyping for Detecting Chromosome Alterations

To establish a method for calling digital karyotypes using the Illumina HumanCoreExome BeadChip, we first calculated the percentage of cells within a population that were required to detect a chromosome alteration. We used the low-quality iPSC sample, CV-hiPS-F (Gore et al., 2011), which was reported by standard G-banding to have trisomy 12, 13, 14, 17, 20, and XXY. The reported frequency of these abnormalities was 90% (18 of 20 cells analyzed

Table 2. Summary of Reprogramming Results

Fibroblast	% GFP$^+$ Cells on Day 2 after Retrovirus Transduction	No. of Colonies	No. of Colonies Picked	No. of Lines that Grew to P3 for Barcoding	No. of Lines Frozen (TRA-1-60$^+$ and TRA-1-81$^+$ with No GFP
APP V717F-1	96.3	>100	49	27	24
APP V717F-2	95.0	>100	32	14	14
NDC4	85.0	36	36	15	14
NDC5	88.3	25	25	20	17
SAD3	76.4	24	24	20	20
SAD4	93.0	>100	44	24	23
SAD5	92.4	>100	34	30	28
SAD6	94.6	>100	50	12	9

by karyotype analysis at WiCell). Using a genetically matched clone of CV-hiPS-F that has a normal karyotype (CV-hiPS-B), we performed a serial dilution of the DNA from CV-hiPS-F (100%, 50%, 25%, 12.5%, 6.25%, 0%) and hybridized the six samples to the HumanCoreExome BeadChip (Figure 5A). These arrays produce data from intensity signals corresponding to the presence of allele A and allele B at a given SNP. Using GenomeStudio (Illumina), we calculated the mean log R ratio (LRR, Tables S4 and S5), a measure of copy number as a ratio of observed to expected intensities; and the B-allele frequency (BAF, Table S6), the proportion of allele calls at each genotype with respect to allele B (1.0 for B/B, 0.5 for A/B, and 0.0 for A/A). We created plots using these metrics to visually inspect each chromosome for abnormalities, and present the findings for chromosomes 13 and 14 in Figure 5A. The BAF and LRR plots depict the disappearance of the trisomic signal of chromosomes 13 and 14 between the 25% and 12.5% dilution samples, indicating that the array is sensitive enough to detect abnormal cells present at about 20% frequency (undiluted DNA has 90% abnormal cells).

To further examine the percentage of abnormal cells detectable by the array, we calculated the mean BAF distance between abnormal (ABB/AAB) and normal (AB) genotypes on the array by filtering the data for the heterozygous SNPs coming from the abnormal autosomes (12, 13, 14, 17, and 20). The signal intensities of the six dilution series datasets were each divided into two clusters based on whether they were above or below the median BAF value. The "AAB" cluster included all SNPs with BAF greater than the median BAF, while the "ABB" cluster had BAF values less than the median BAF. The mean BAF value was then calculated for the ABB and AAB groups; the difference between these values yielded the BAF mean distance. A high BAF mean distance signifies a clear signature for a trisomy, whereas a lower value signifies normal diploid DNA. These distances were then plotted (Figure 5B), providing additional evidence that the array can detect abnormal cells that are present at a 20% or greater frequency (between the 25% and 12.5% dilution samples). This observed detection sensitivity shows that SNP arrays can detect possibly harmful alterations in small subpopulations of iPSC lines.

DISCUSSION

Here we provide a workflow that enables rapid and cost-effective characterization of iPSC lines. Our suite of methods combines FCB, 12-gene qPCR, and SNP arrays to measure heterogeneity, expression levels of differentiation genes, and chromosomal aberrations, respectively. The FCB method can substantially reduce the labor and time required for performing flow cytometry as up to 60 iPSC samples can be processed simultaneously (Figure 1). Because FCB is conducted in a 96-well format, it can significantly reduce antibody consumption and eliminate staining variability between samples. The high-throughput nature of FCB and the fact that the only instrument required is a flow cytometer, which is present in most research facilities, allows it to be readily incorporated into workflows for characterizing the pluripotency of large-scale iPSC collections on a per-line cost basis of ~$12 (based on cost of $1,200 to purchase all dyes for barcoding, and TRA-1-60 and TRA-1-81 antibodies, which can be used to label 100 samples).

The qPCR method we describe uses the expression levels of only 12 genes to provide a qualitative assessment of each of the three germ layers (four genes each), which is a cost-effective and high-throughput approach to rapidly assess the pluripotency and basic differentiation potential of iPSCs. Although we used a Fluidgm Biomark HD instrument (full Biomark chip costs ~$1,500 to run with reagents

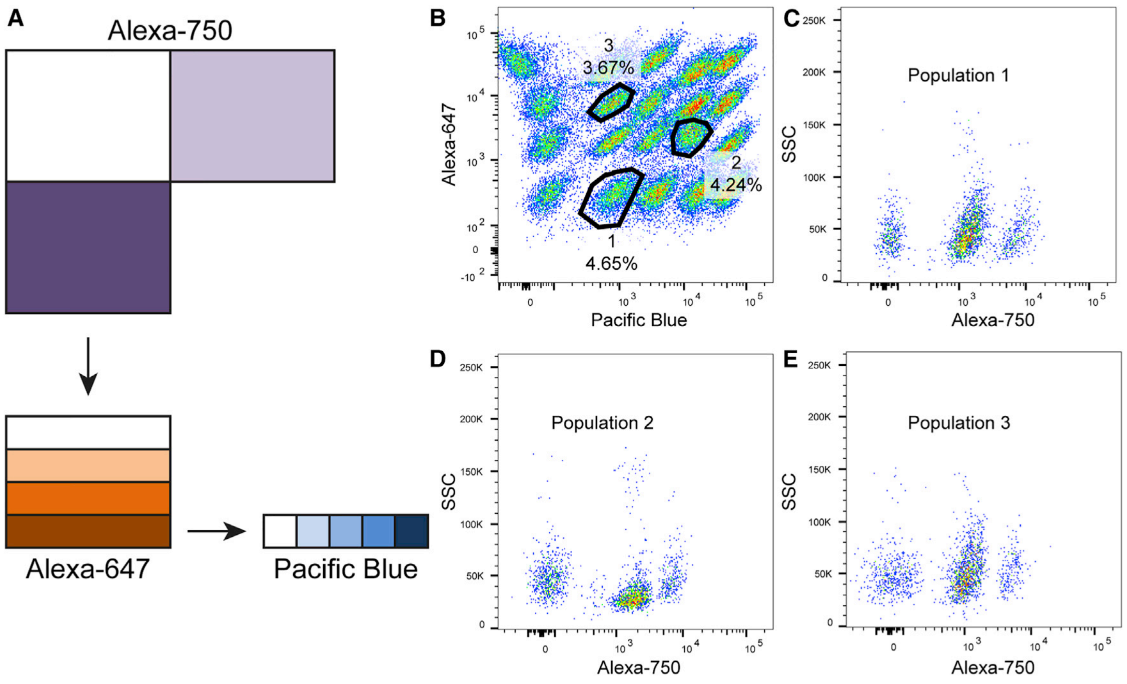

Figure 2. Optimization of Fluorescent Cell Barcoding Technique for Use with iPSCs

(A) FCB setup for barcoding 60 iPSC samples using three dyes: Alexa 750, Alexa 647, and Pacific Blue. FCB was optimized to include three concentrations of Alexa 750 (0, 5, 15 μg/mL), four concentrations of Alexa 647 (0, 0.6, 3, 15 μg/mL), and five concentrations of Pacific Blue (0, 0.4, 1.6, 6.4, 25 μg/mL).

(B) Efficient barcoding of iPSCs with Alexa 647 and Pacific Blue allows clear distinction of 20 iPSC populations stained with different concentrations of these two dyes.

(C–E) The three populations indicated in (B) are each deconvoluted into three distinct iPSC lines based on staining with Alexa 750. SSC, side-scattered light.

and labor) to develop our approach, standard qPCR instruments that are present in most research facilities can be utilized to run the 12-gene qPCR on a per-line cost basis of ~$20 (based on a cost of ~$100 to purchase IDT PrimeTime qPCR Assay and ~$70 for Probe-Based qPCR Master Mix which can be used for 100 samples). This enables the simultaneous interrogation of hundreds of samples for the expression of markers for each of the three germ layers (Figure 1) at substantially less cost than for available tests (such as the commercially available ScoreCard) (Tsankov et al., 2015). In Data S1, we provide an Excel spreadsheet that allows the calculation of pluripotency and germ layer scores for those interested in implementing our 12-gene qPCR approach.

Using digital karyotyping by SNP arrays, the genomic integrity of iPSC lines can be initially examined and also easily monitored at different passages over time, which is recommended due to genomic changes that can occur as cells remain in culture, but in practice is not done with conventional karyotyping due to costs. Importantly, we showed that SNP arrays can be used to detect both clonal (present in all iPSC cells) and subclonal (present in ~20% or more cells) CNVs, suggesting that digital karyotyping is a useful method to detect potentially harmful genomic alterations that are present only in a subpopulation of iPSCs within a line. While a full SNP array (24 individual samples) (Figure 1) can cost $1,800 with reagent and labor costs, on a per-line cost basis it is ~$75 ($1,800/24). Thus, research facilities with Illumina microarray scanners can readily implement digital karyotyping. (For researchers without access to Illumina machines, samples may be sent to our facility at University of California at San Diego [UCSD]; see Experimental Procedures). In summary, our suite of methods provides excellent characterization of the heterogeneity, pluripotency, and genomic integrity of an iPSC line for ~$110.

EXPERIMENTAL PROCEDURES

iPSC Generation

iPSCs were generated as previously described (Israel et al., 2012) with a few minor modifications. Each transcription factor vector contained a GFP tag to allow for monitoring of silencing of retroviral factors (Chan et al., 2009). iPSCs were maintained on an

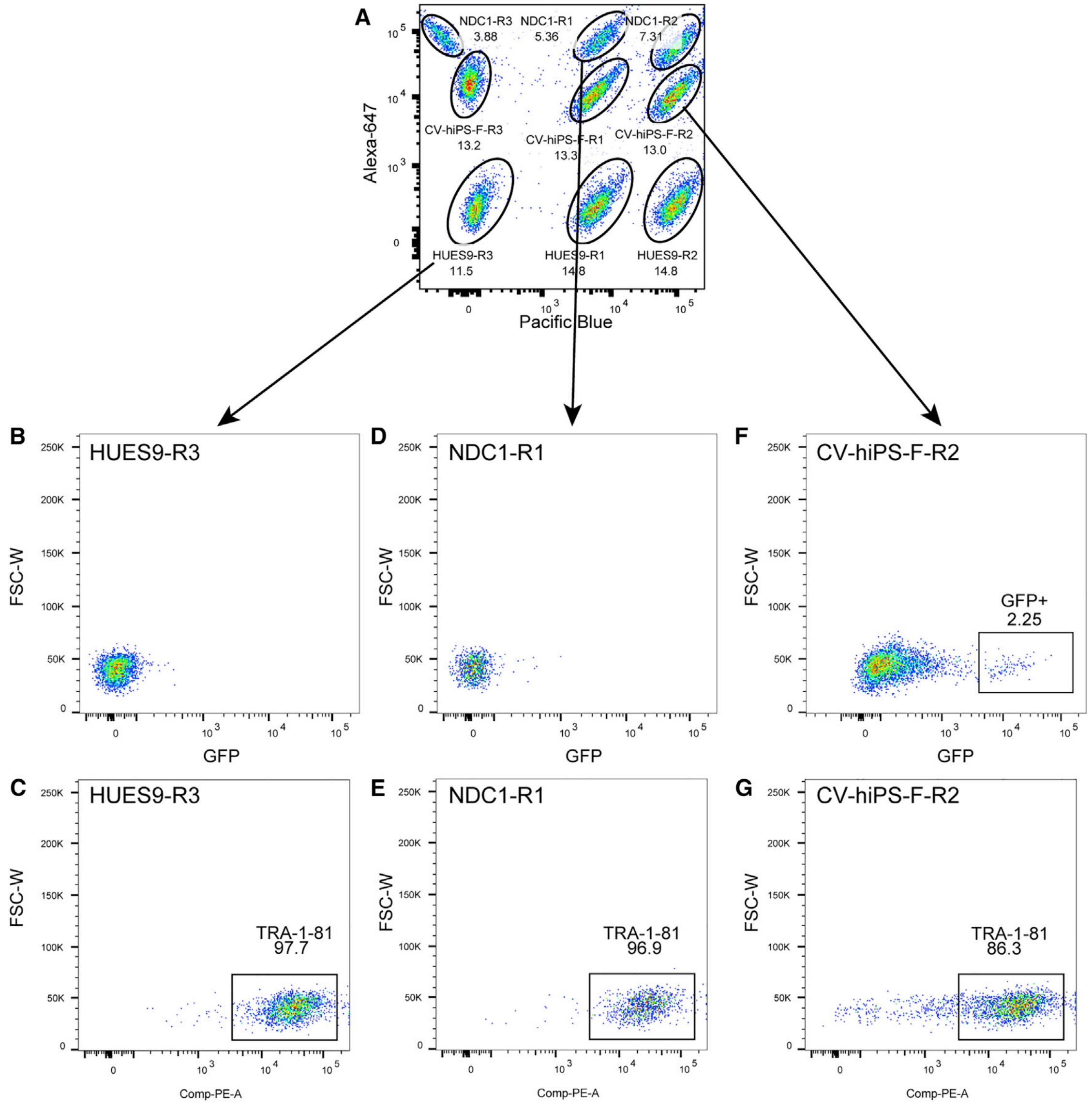

Figure 3. Fluorescent Cell Barcoding Can Distinguish between High- and Low-Quality iPSCs

(A) One hESC line (HUES9), one high-quality iPSC line (NDC1), and one low-quality iPSC line (CV-hiPS-F) were barcoded, stained with TRA-1-81 and TRA-1-60 separately, and subsequently analyzed for GFP expression in three replicates (depicted as R1, R2, R3). Only data for staining with TRA-1-81 are shown.

(B and C) The HUES9-R3 hESC (B) displays no GFP$^+$ cells and (C) has 97.7% of cells TRA-1-81$^+$.

(D and E) The NDC1-R1 high-quality iPSC line (D) displays no GFP$^+$ cells and (E) has 96.9% of cells TRA-1-81$^+$.

(F and G) The CV-hiPS-F-R2 low-quality iPSC line has (F) GFP$^+$ cells, indicating retrovirus reactivation and (G) a lower fraction of TRA-1-81$^+$ cells (86.3%).

FSC-W, forward-scattered light width. See also Figure S1.

irradiated murine embryonic fibroblast (MEF) feeder layer with medium containing knockout (KO) DMEM (Gibco), 20% KO Serum Replacement (Gibco), 20 mM GlutaMax (Invitrogen), 20 mM non-essential amino acids (Invitrogen), 20 mM penicillin-streptomycin (Invitrogen), and 20 ng/mL fibroblast growth factor (FGF) (Millipore). Cells were passaged by dissociation with Accutase (Innovative Cell Technologies).

Fluorescent Cell Barcoding

FCB was performed as previously described (Krutzik and Nolan, 2006). In brief, in a 96-well format cells were fixed for 10 min at room temperature in 4% paraformaldehyde. Following fixation, cells were resuspended in 100% methanol with fluorescent dyes (Life Technologies) and incubated at room temperature for 20 min. Cells were subsequently washed twice in PBS containing

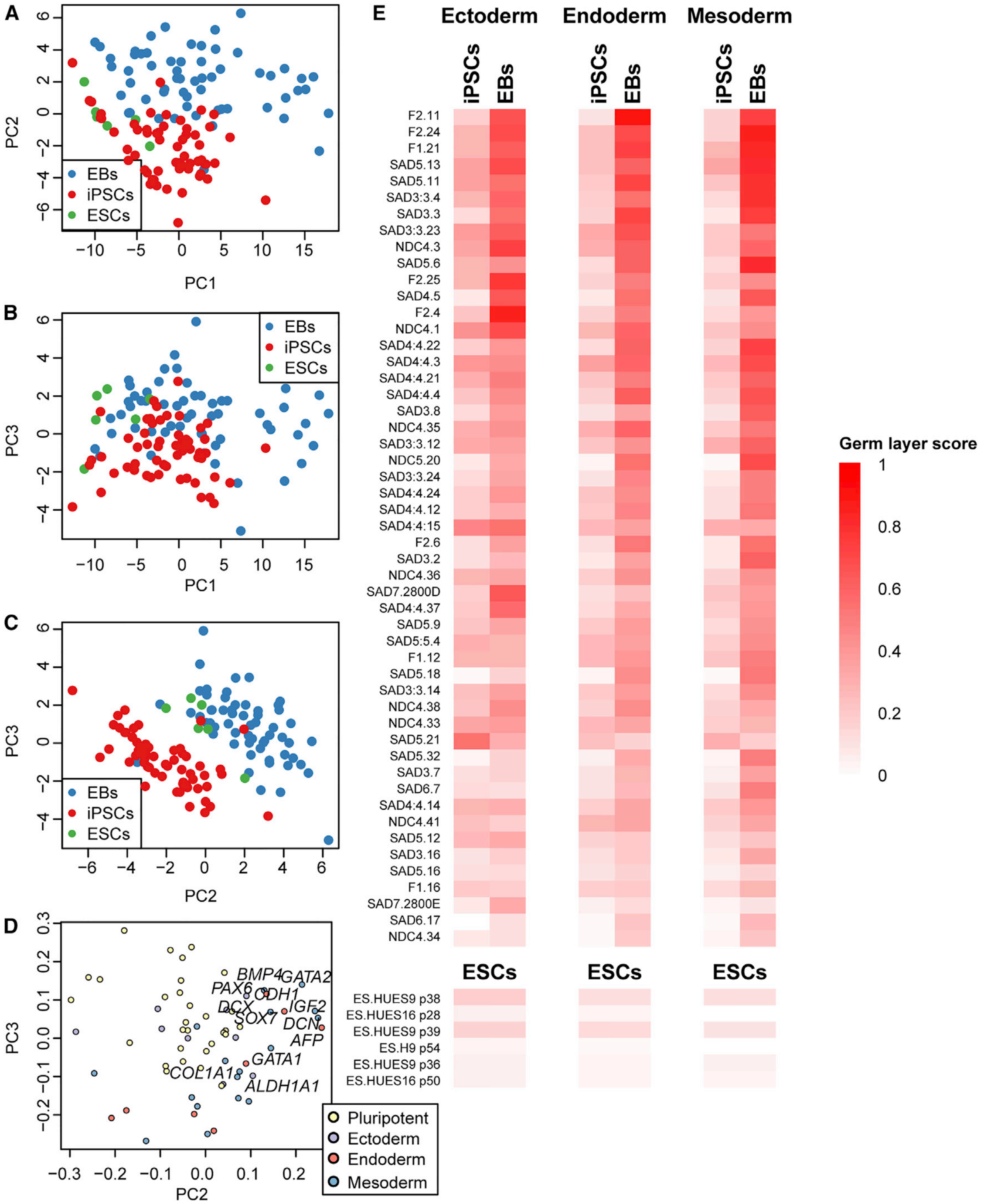

Figure 4. Germ Layer Scores Show Expression Differences between iPSCs and EBs

(A–C) Principal component analysis of expression of 64 marker genes as measured by qPCR using the Fluidigm Biomark platform: (A) scatterplot PC1-PC2; (B) scatterplot PC1-PC3; (C) scatterplot PC2-PC3. The three plots show that the expression values of the 64 marker genes are able to distinguish iPSCs from embryoid bodies (EBs).

(D) Scatterplot of the weights of each gene on PC2 (x axis) and PC3 (y axis). The 12 genes selected for the qPCR are shown (and are also listed in Table S2). Four genes for each germ layer were chosen because they contributed to the largest expression differences between iPSCs and EBs.

(E) Germ layer scores for iPSCs and EBs (top) and ESCs (bottom) were calculated as the mean value across the four genes in each set. The majority of EBs display high scores for all three germ layers, whereas iPSCs and ESCs have low scores.

See also Tables S1–S3 and Data S1.

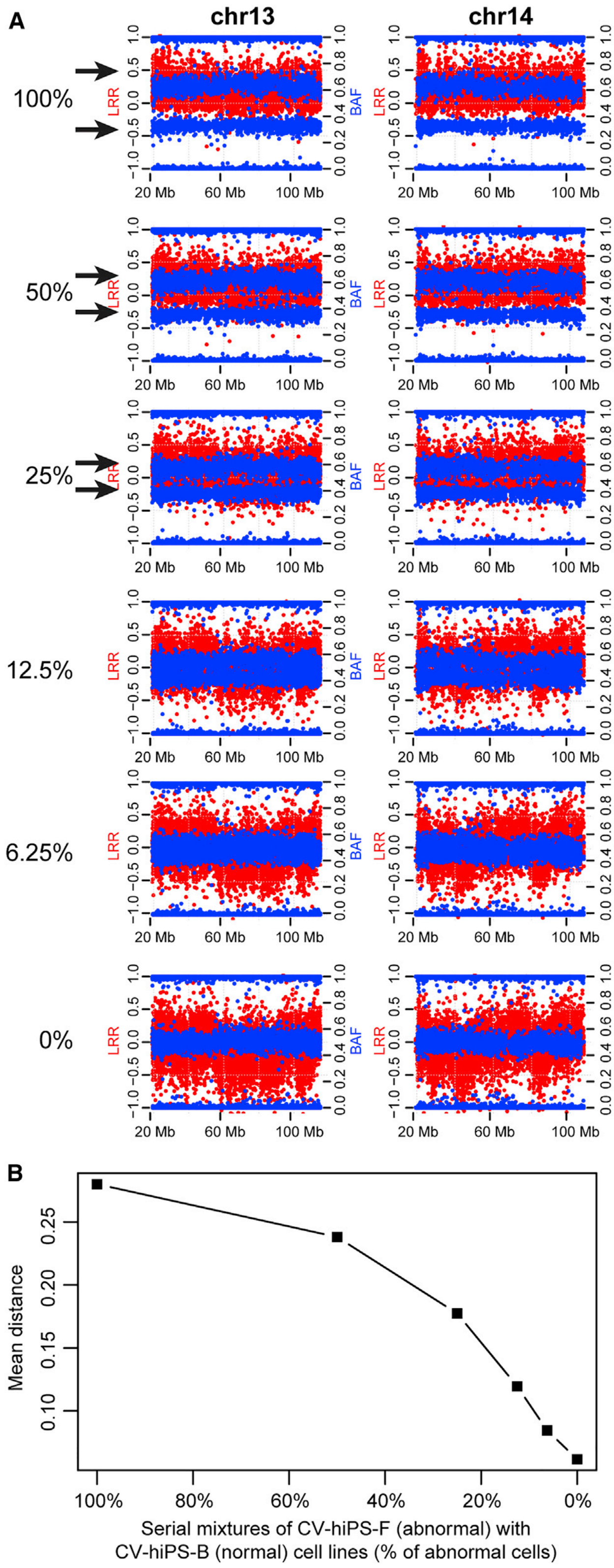

Figure 5. Serial Dilution of an Abnormal iPSC Line to Establish Detection Sensitivity

(A) Mean log R ratio (LRR, in red) and B-allele frequency (BAF, in blue) in the six dilution states (0%, 6.25%, 12.5%, 25%, 50%, and 100%). Notably, the AAB/ABB trisomy specific BAF signal (the two blue horizontal bands) transitions to the diploid AB BAF signal (single blue band) as the amount of abnormal cell line in the assay decreases. The black arrows highlight the heterozygous SNP band as it changes from the AAB and ABB genotype (two blue bands) to the AB genotype (one blue band). The disappearance of the double-band AAB/ABB genotype to the single-band AB genotype indicates the failure of the assay to detect the difference between abnormal trisomic and normal diploid DNA, and thus we estimate the sensitivity of the array to be between 12.5% and 25%.

(B) Plot displaying the mean BAF distance for chromosomes 12, 13, 14, 17, and 20 across the six dilution states. High BAF mean distance indicates a trisomic state, while a lower value signifies normal diploid DNA.

See also Tables S4–S6.

0.1% BSA. Barcoded cells where then combined together and divided into two tubes: one tube of cells was stained with TRA-1-60 and the other was stained with TRA-1-81 (BD Biosciences, 1:1,000). Barcoded samples were then measured by flow cytometry (BD Biosciences) and all data was analyzed using FlowJo Cell Analysis software. Only cells expressing high levels of both markers and that were GFP negative were used in qPCR analysis.

Embryoid Body Generation

EBs were generated as previously described (Bock et al., 2011). In brief, iPSCs were lifted off the MEFs using dispase and were then plated in low-attachment plates in the presence of IPSC culture medium without FGF. EBs were grown for 2 weeks and the medium was changed every 48 hr.

qPCR Analysis

Primers were designed for 68 genes (4 housekeeping genes and 64 pluripotency or germ layer markers) using an in-house algorithm that targeted: (1) a melting temperature of 60°C; (2) regions that spanned multiple exons when possible to minimize genomic DNA signal contamination; and (3) exons that are shared between multiple isoforms. In addition, the primers were tested and showed a single dominant melting curve peak consistent with a dominant amplification product (see Table S2 for a list of primers).

To select the most consistently expressed housekeeping gene for normalization, we tested four housekeeping genes (*RPS29*, *GAPDH*, *RPL22*, and *DSG2*). We selected *RPS29* because this gene had the least variable Ct distribution across all samples (SD = 0.827), whereas the other three housekeeping genes had higher variability (SD = 1.629, 1.623, and 1.642, respectively). The exclusion of these three housekeeping genes resulted in the expression of 65 genes being utilized (1 housekeeping and 64 marker genes).

RNA was isolated from samples using either TRIzol, Qiagen RNeasy Mini Kit, or Qiagen AllPrep DNA/RNA kit. cDNA was produced using Superscript III from 100–500 ng input RNA using oligo(dT)20. Samples were prepared following the Fluidigm Advanced Development Protocol v37, and qPCR was performed

on the Fluidigm BioMark HD using EvaGreen and the GE96x96 Fast PCR protocol.

Marker Gene Selection for 12-Gene qPCR

Gene expression (Ct) was normalized to *RPS29* and used as input for PCA. PCA was performed using the prcomp function in R and PCs were visually inspected (Figure 4), with PC2 and PC3 found to have the largest differences between iPSCs and EBs. EBs were found to be associated with high values of PC2 and PC3, whereas iPSCs had low values of PC2 and PC3. To calculate a "germ layer score," four genes per germ layer were chosen to be included because they had the most negative weights on PC2 and PC3. Expression levels for all genes were normalized across all 110 samples in order to have minimum = 0 and maximum = 1, and the germ layer scores were calculated as the mean value across each group of four genes. We include a supplementary file, Data S1, a Microsoft Excel table that allows users to generate germ layer scores using Ct values as input. Similarly, the user can use Data S1 to generate pluripotency scores using Ct values for four pluripotency markers (chosen by the user) as input.

HumanCoreExome BeadChips

Using the services of the UCSD IGM Genomics Center (http://igm.ucsd.edu/genomics/), genomic DNA from a normal iPSC line (CV-hiPS-B) and a genetically identical abnormal iPSC line (CV-hiPS-F) were extracted (AllPrep DNA/RNA Mini Kit, Qiagen), normalized to 200 ng, serially diluted, hybridized to HumanCoreExome v12 arrays (Illumina), and stained and scanned using the Illumina HiScan system per standard protocol. We observed an average call rate of 99.2% across the arrays.

The Institutional Review Board of the University of California at San Diego approved the study and the subject whose DNA was used for the HumanCoreExome BeadChips gave informed consent (Project #071641).

ACCESSION NUMBERS

The accession number for the HumanCoreExome BeadChip data reported in this paper is ArrayExpress: E-MTAB-5587.

SUPPLEMENTAL INFORMATION

Supplemental Information includes Supplemental Experimental Procedures, one figure, six tables, and one data file and can be found with this article online at http://dx.doi.org/10.1016/j.stemcr.2017.03.011.

AUTHOR CONTRIBUTIONS

M.D. performed statistical analyses. A.A., H.M., A.D.P., and R.W. performed and analyzed HumanCoreExome arrays. G.W. and C.H. reprogrammed fibroblasts and generated EBs. G.W. and S.M.R. optimized the FCB protocol and G.W. performed FCB on reprogrammed lines. A.D.-C. performed flow cytometry analysis. J.L.N. performed qPCR. K.A.F., G.W.Y., and L.S.B.G. oversaw the study. A.D.P., M.D., K.A.F., G.W.Y., and L.S.B.G. wrote the manuscript.

ACKNOWLEDGMENTS

This work was supported in part by a California Institute for Regenerative Medicine (CIRM) grant GC1R-06673 and NIH grants HG008118-01, HL107442-05, DK105541, DK112155, and EY021237 to K.A.F. This work was also supported by grants from the NIH (NS075449 and HG004659) to G.W.Y. and the CIRM grant RT2-01927 to L.S.B.G. and G.W.Y. HumanCoreExome BeadChip data generation were performed at the UCSD IGM Genomics Center with support from NIH grant P30CA023100. We thank the Gallagher family for their generous support of stem cell research at the University of Notre Dame.

Received: December 7, 2016
Revised: March 10, 2017
Accepted: March 13, 2017
Published: April 6, 2017

REFERENCES

Andrews, P.W., Baker, D., Benvinisty, N., Miranda, B., Bruce, K., Brustle, O., Choi, M., Choi, Y.M., Crook, J.M., de Sousa, P.A., et al. (2015). Points to consider in the development of seed stocks of pluripotent stem cells for clinical applications: International Stem Cell Banking Initiative (ISCBI). Regen. Med. *10*, 1–44.

Avior, Y., Sagi, I., and Benvenisty, N. (2016). Pluripotent stem cells in disease modelling and drug discovery. Nat. Rev. Mol. Cell Biol. *17*, 170–182.

Bock, C., Kiskinis, E., Verstappen, G., Gu, H., Boulting, G., Smith, Z.D., Ziller, M., Croft, G.F., Amoroso, M.W., Oakley, D.H., et al. (2011). Reference Maps of human ES and iPS cell variation enable high-throughput characterization of pluripotent cell lines. Cell *144*, 439–452.

Brennand, K.J., Simone, A., Jou, J., Gelboin-Burkhart, C., Tran, N., Sangar, S., Li, Y., Mu, Y., Chen, G., Yu, D., et al. (2011). Modelling schizophrenia using human induced pluripotent stem cells. Nature *473*, 221–225.

Chan, E.M., Ratanasirintrawoot, S., Park, I.H., Manos, P.D., Loh, Y.H., Huo, H., Miller, J.D., Hartung, O., Rho, J., Ince, T.A., et al. (2009). Live cell imaging distinguishes bona fide human iPS cells from partially reprogrammed cells. Nat. Biotechnol. *27*, 1033–1037.

Elliott, A.M., Elliott, K.A., and Kammesheidt, A. (2010). High resolution array-CGH characterization of human stem cells using a stem cell focused microarray. Mol. Biotechnol. *46*, 234–242.

Folstein, M.F., Folstein, S.E., and McHugh, P.R. (1975). "Mini-mental state". A practical method for grading the cognitive state of patients for the clinician. J. Psychiatr. Res. *12*, 189–198.

Gore, A., Li, Z., Fung, H.L., Young, J.E., Agarwal, S., Antosiewicz-Bourget, J., Canto, I., Giorgetti, A., Israel, M.A., Kiskinis, E., et al. (2011). Somatic coding mutations in human induced pluripotent stem cells. Nature *471*, 63–67.

Hulten, M.A., Dhanjal, S., and Pertl, B. (2003). Rapid and simple prenatal diagnosis of common chromosome disorders: advantages and disadvantages of the molecular methods FISH and QF-PCR. Reproduction *126*, 279–297.

International Stem Cell Initiative, Amps, K., Andrews, P.W., Anyfantis, G., Armstrong, L., Avery, S., Baharvand, H., Baker, J., Baker, D., Munoz, M.B., et al. (2011). Screening ethnically diverse human embryonic stem cells identifies a chromosome 20 minimal amplicon conferring growth advantage. Nat. Biotechnol. *29*, 1132–1144.

Israel, M.A., Yuan, S.H., Bardy, C., Reyna, S.M., Mu, Y., Herrera, C., Hefferan, M.P., Van Gorp, S., Nazor, K.L., Boscolo, F.S., et al. (2012). Probing sporadic and familial Alzheimer's disease using induced pluripotent stem cells. Nature *482*, 216–220.

Itzhaki, I., Maizels, L., Huber, I., Zwi-Dantsis, L., Caspi, O., Winterstern, A., Feldman, O., Gepstein, A., Arbel, G., Hammerman, H., et al. (2011). Modelling the long QT syndrome with induced pluripotent stem cells. Nature *471*, 225–229.

Kilpinen, H., Goncalves, A., Leha, A., Afzal, V., Ashford, S., Bala, S., Bensaddek, D., Casale, F.P., Culley, O., Danacek, P., et al. (2016). Common genetic variation drives molecular heterogeneity in human iPSCs. bioRxiv http://dx.doi.org/10.1101/055160.

Krutzik, P.O., and Nolan, G.P. (2006). Fluorescent cell barcoding in flow cytometry allows high-throughput drug screening and signaling profiling. Nat. Methods *3*, 361–368.

Kurosawa, H. (2007). Methods for inducing embryoid body formation: in vitro differentiation system of embryonic stem cells. J. Biosci. Bioeng. *103*, 389–398.

Laurent, L.C., Ulitsky, I., Slavin, I., Tran, H., Schork, A., Morey, R., Lynch, C., Harness, J.V., Lee, S., Barrero, M.J., et al. (2011). Dynamic changes in the copy number of pluripotency and cell proliferation genes in human ESCs and iPSCs during reprogramming and time in culture. Cell Stem Cell *8*, 106–118.

Maitra, A., Arking, D.E., Shivapurkar, N., Ikeda, M., Stastny, V., Kassauei, K., Sui, G., Cutler, D.J., Liu, Y., Brimble, S.N., et al. (2005). Genomic alterations in cultured human embryonic stem cells. Nat. Genet. *37*, 1099–1103.

Mayshar, Y., Ben-David, U., Lavon, N., Biancotti, J.C., Yakir, B., Clark, A.T., Plath, K., Lowry, W.E., and Benvenisty, N. (2010). Identification and classification of chromosomal aberrations in human induced pluripotent stem cells. Cell Stem Cell *7*, 521–531.

McKernan, R., and Watt, F.M. (2013). What is the point of large-scale collections of human induced pluripotent stem cells? Nat. Biotechnol. *31*, 875–877.

Mertens, J., Wang, Q.W., Kim, Y., Yu, D.X., Pham, S., Yang, B., Zheng, Y., Diffenderfer, K.E., Zhang, J., Soltani, S., et al. (2015). Differential responses to lithium in hyperexcitable neurons from patients with bipolar disorder. Nature *527*, 95–99.

Muller, F.J., Schuldt, B.M., Williams, R., Mason, D., Altun, G., Papapetrou, E.P., Danner, S., Goldmann, J.E., Herbst, A., Schmidt, N.O., et al. (2011). A bioinformatic assay for pluripotency in human cells. Nat. Methods *8*, 315–317.

Panopoulos, A.D., D'Antonio, M., Benaglio, P., Williams, R., Hashem, S.I., Schuldt, B.M., DeBoever, C., Arias, A.D., Garcia, M., Nelson, B., et al. (2017). iPSCORE: a resource of 222 iPSC lines enabling functional characterization of genetic variation across a variety of cell types. Stem Cell Rep. *8*, this issue, 1086–1100.

Salomonis, N., Dexheimer, P.J., Omberg, L., Schroll, R., Bush, S., Huo, J., Schriml, L., Ho Sui, S., Keddache, M., Mayhew, C., et al. (2016). Integrated genomic analysis of diverse induced pluripotent stem cells from the progenitor cell biology consortium. Stem Cell Rep. *7*, 110–125.

Spits, C., Mateizel, I., Geens, M., Mertzanidou, A., Staessen, C., Vandeskelde, Y., Van der Elst, J., Liebaers, I., and Sermon, K. (2008). Recurrent chromosomal abnormalities in human embryonic stem cells. Nat. Biotechnol. *26*, 1361–1363.

Taapken, S.M., Nisler, B.S., Newton, M.A., Sampsell-Barron, T.L., Leonhard, K.A., McIntire, E.M., and Montgomery, K.D. (2011). Karotypic abnormalities in human induced pluripotent stem cells and embryonic stem cells. Nat. Biotechnol. *29*, 313–314.

Tsankov, A.M., Akopian, V., Pop, R., Chetty, S., Gifford, C.A., Daheron, L., Tsankova, N.M., and Meissner, A. (2015). A qPCR ScoreCard quantifies the differentiation potential of human pluripotent stem cells. Nat. Biotechnol. *33*, 1182–1192.

Wapner, R.J., Martin, C.L., Levy, B., Ballif, B.C., Eng, C.M., Zachary, J.M., Savage, M., Platt, L.D., Saltzman, D., Grobman, W.A., et al. (2012). Chromosomal microarray versus karyotyping for prenatal diagnosis. N. Engl. J. Med. *367*, 2175–2184.

Wu, H., Kim, K.J., Mehta, K., Paxia, S., Sundstrom, A., Anantharaman, T., Kuraishy, A.I., Doan, T., Ghosh, J., Pyle, A.D., et al. (2008). Copy number variant analysis of human embryonic stem cells. Stem Cells *26*, 1484–1489.

Stem Cell Reports, Volume *8*

Supplemental Information

High-Throughput and Cost-Effective Characterization of Induced Pluripotent Stem Cells

Matteo D'Antonio, Grace Woodruff, Jason L. Nathanson, Agnieszka D'Antonio-Chronowska, Angelo Arias, Hiroko Matsui, Roy Williams, Cheryl Herrera, Sol M. Reyna, Gene W. Yeo, Lawrence S.B. Goldstein, Athanasia D. Panopoulos, and Kelly A. Frazer

Supplemental information includes Figure S1, Tables S1-S6 and Data S1.

Supplemental Figure

Figure S1. Differences between low- and high-quality barcoded iPSCs, related to Figure 3.

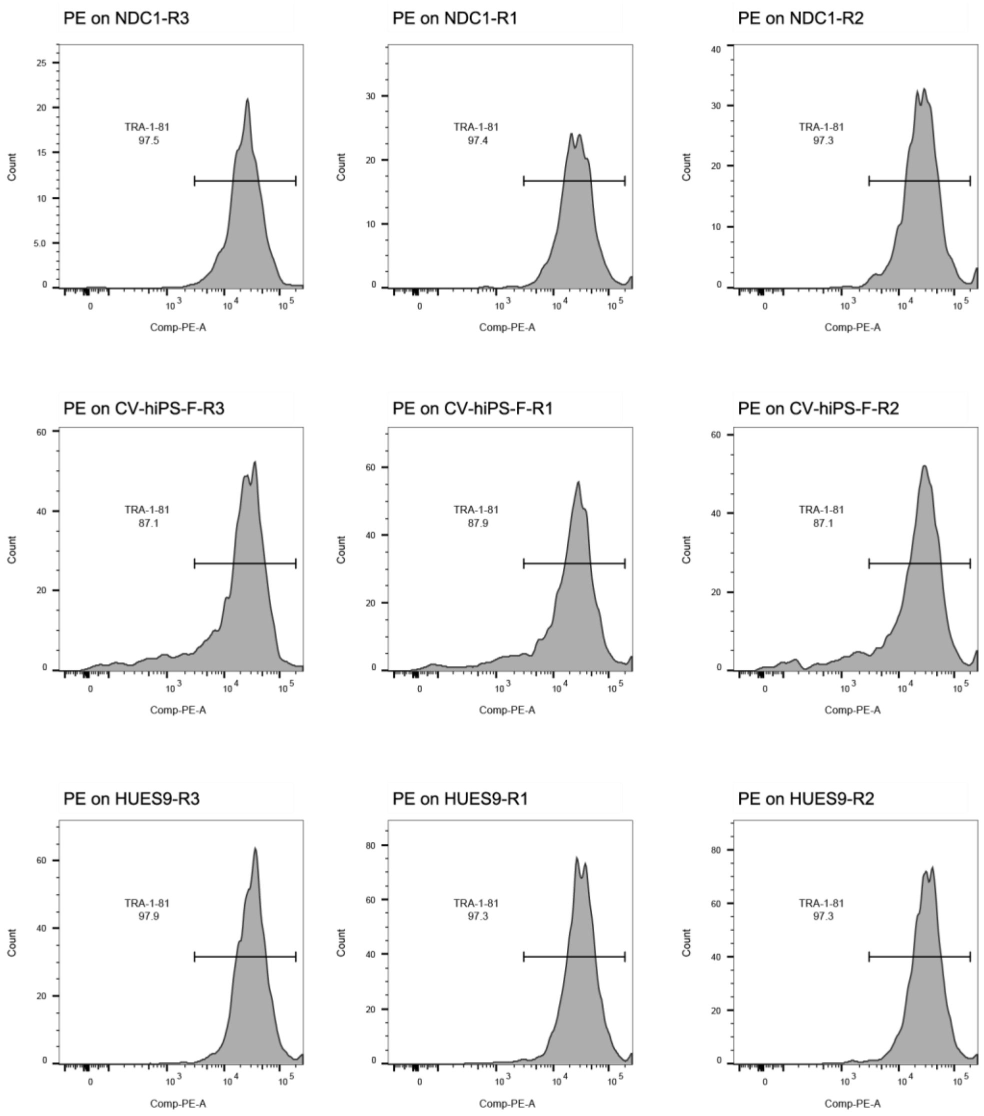

Shown are the histograms of TRA-1-81^{+} cells for the three replicates of the three lines analyzed in Figure 3. A lower percentage of cells staining positive for TRA-1-81^{+} in the CV-hiPS-F line (middle row) is in agreement with previous characterization of its quality (Gore et al., 2011), and confirms that FCB allows the distinction between low- and high-quality iPSCs.

Supplemental Tables

Table S1. Description of all lines used for qPCR, related to Figure 4.

The table shows the list of 122 lines used for qPCR. Column C shows the 12 samples that did not pass QC (five iPSCs and one EB, as well as their associated five EBs and one iPSC line) and were not included in PCA (Figure 4).

Table S2. List of primers used for qPCR, related to Figure 4.

Shown is the list of all 68 primer pairs (4 housekeeping genes and 64 pluripotency or germ layer markers) used for qPCR. For each primer pair, the lineage where its associated gene is expressed is displayed (column B). Column C shows the 12 primer pairs that were selected for 12-gene qPCR to calculate each germ layer scores (Figure 4).

Table S3. Ct values detected by qPCR, related to Figure 4.

Shown are the raw Ct values of all 68 primer pairs in the 110 samples that underwent qPCR and passed all quality filters and were used for PCA.

Table S4. Genomic position of all variants in trisomic chromosomes, related to Figure 5.

Shown is the probe ID assigned by Illumina and the genomic position for all 50,076 probes in the trisomic chromosomes (12, 13, 14, 17, 20).

Table S5. Log r ratio for all variants in trisomic chromosomes, related to Figure 5.

Shown is the log r ratio associated with the probes described in Table S4 for CV-hiPS-B, CV-hiPS-F and the serial dilutions.

Table S6. B allele frequency for all variants in trisomic chromosomes, related to Figure 5.

Shown is the B allele frequency associated with the probes described in Table S4 for CV-hiPS-B, CV-hiPS-F and the serial dilutions.

Supplemental Data

Data S1. Calculation of pluripotency and germ layer scores, related to Figure 4.

This Excel sheet allows the user to calculate pluripotency and germ layer scores by providing raw Ct values of 17 genes (four pluripotency markers, four markers for each germ layer and one housekeeping gene) as input. The 12 genes for the three germ layer scores were chosen based on analyses in this manuscript (Figure 4). The four pluripotency markers are from our previous publication (Gore et al., 2011; Liu et al., 2011).

To run the analysis, users will need to: 1) enter sample names in cells A4-A99 (highlighted in blue); 2) enter gene names in cells B3-R3 (highlighted in blue); 3) enter the raw Ct values for the housekeeping gene to be used for normalization in cells B4-B99 (highlighted in green); and 4) enter raw Ct values for the marker genes in cells C4-R99 (highlighted in yellow).

Columns T-AJ show ΔCt values, calculated as $\Delta Ct_{gene} = 2^{(Ct_{housekeeping} - Ct_{gene})}$. Columns AL-BB show normalized expression levels (to values included between zero and one), calculated as $e_{gene} = \frac{(\Delta Ct_{gene} - \min(\Delta Ct_{gene}))}{(\max(\Delta Ct_{gene}) - \min(\Delta Ct_{gene}))}$. The output scores are shown in cells BD2-BH99, while the corresponding heatmap is shown in cells BJ2-BN99.

For demonstrative purposes, two example datasets are provided for the user to test the Data S1 Excel sheet. Dataset 1 is provided to test calculating a pluripotency score, the Excel sheet is populated with raw Ct values from five samples (including two ESCs, two iPSCs and one fibroblast) that have been previously characterized (Gore et al., 2011; Liu et al., 2011). Dataset 2 is provided to test germ-layer scores, the Excel sheet is populated with raw Ct values from nine samples (three ESCs, three iPSCs and their associated EBs) generated in this manuscript.

Below are the two example datasets provided for the user to test the Data S1 Excel sheet:

Dataset 1: Five samples assessed for four pluripotency markers (Gore et al., 2011; Liu et al., 2011). The user pastes these sample names, gene names and raw Ct values of each gene into Data S1 Excel sheet.

**Sample name**	**Housekeeping gene**	**Pluripotent**			
	**GAPDH**	**End-OCT4**	**End-SOX2**	**NANOG**	**CRIPTO**
H1 (ES Line)	14.500	18.820	18.000	19.000	17.000
H9 (ES line)	14.670	17.000	18.000	19.000	17.000
FiPS4F2 (iPS line)	13.950	18.000	18.000	20.000	17.000
FiPS4F5 (iPS line)	15.860	19.000	20.000	20.000	18.000
IMR90 (Fibroblasts)	14.500	23.000	31.000	27.000	29.000

Dataset 2: Nine samples with 12 germ layer markers (four for each germ layer). The user pastes these sample names, gene names and raw Ct values of each gene into Data S1 Excel sheet.

Sample name	Housekeeping gene	Ectoderm			
	RPS29	ALDH1A1	COL1A1	PAX6	DCX
ES.HUES16 p50	2.500	13.310	8.925	13.901	14.302
ES.HUES9 p39	3.364	15.790	10.500	20.077	14.460
ES.H9 p54	2.500	14.285	11.136	16.280	13.530
IPS.F2.11	3.146	14.796	9.321	13.168	15.250
IPS.F2.24	3.811	16.486	8.934	14.849	15.706
IPS.F1.21	3.728	15.662	8.790	14.514	15.975
EB.F1.21	4.771	13.870	8.422	15.564	15.442
EB.F2.11	4.537	11.970	8.011	14.046	15.820
EB.F2.24	5.323	14.977	9.229	15.074	17.497

Sample name	Housekeeping gene	Endoderm			
	RPS29	AFP	GATA1	SOX7	CDH1
ES.HUES16 p50	2.500	13.320	20.451	16.000	12.768
ES.HUES9 p39	3.364	25.800	20.192	16.588	13.490
ES.H9 p54	2.500	999.000	23.679	14.778	12.333
IPS.F2.11	3.146	16.445	999.000	16.368	12.778
IPS.F2.24	3.811	18.758	21.379	17.129	12.580
IPS.F1.21	3.728	13.694	21.679	17.002	12.999
EB.F1.21	4.771	5.948	20.712	14.518	12.844
EB.F2.11	4.537	3.869	16.835	13.737	11.062
EB.F2.24	5.323	6.852	999.000	14.907	11.805

Sample name	Housekeeping gene	Mesoderm			
	RPS29	DCN	GATA2	BMP4	IGF2
ES.HUES16 p50	2.500	14.114	15.979	11.766	15.107
ES.HUES9 p39	3.364	21.549	17.743	13.597	17.962
ES.H9 p54	2.500	20.024	17.257	13.560	19.049
IPS.F2.11	3.146	13.128	16.635	12.154	13.227
IPS.F2.24	3.811	23.982	17.141	13.781	16.442
IPS.F1.21	3.728	19.900	14.740	11.410	14.250
EB.F1.21	4.771	9.046	13.037	12.312	8.908
EB.F2.11	4.537	9.188	12.863	12.255	9.764
EB.F2.24	5.323	10.275	12.584	13.954	10.119

References

Gore, A., Li, Z., Fung, H.L., Young, J.E., Agarwal, S., Antosiewicz-Bourget, J., Canto, I., Giorgetti, A., Israel, M.A., Kiskinis, E., *et al.* (2011). Somatic coding mutations in human induced pluripotent stem cells. Nature *471*, 63-67.

Liu, G.H., Barkho, B.Z., Ruiz, S., Diep, D., Qu, J., Yang, S.L., Panopoulos, A.D., Suzuki, K., Kurian, L., Walsh, C., *et al.* (2011). Recapitulation of premature ageing with iPSCs from Hutchinson-Gilford progeria syndrome. Nature *472*, 221-225.
